# Respiratory Symptoms among Adolescents in Poland: A Study on Cigarette Smokers, E-Cigarette Users, and Dual Users

**DOI:** 10.3390/pediatric16030044

**Published:** 2024-06-21

**Authors:** Paulina Kurdyś-Bykowska, Leon Kośmider, Dawid Konwant, Krystyna Stencel-Gabriel

**Affiliations:** 1 Department and Clinical Department of Pediatrics in Bytom, Medical University of Silesia, 40-055 Katowice, Poland; 2 Department of General and Inorganic Chemistry, Faculty of Pharmaceutical Sciences in Sosnowiec, Medical University of Silesia in Katowice, 41-200 Sosnowiec, Poland

**Keywords:** adolescent, children, electronic nicotine delivery systems, pulmonary symptoms, e-cigarettes

## Abstract

In recent years, the prevalence of tobacco and electronic cigarette (e-cigarette) use among adolescents has raised significant public health concerns worldwide. This study aimed to investigate respiratory symptoms among Polish adolescents. We conducted an online survey among Polish school students from all provinces, collecting data over two months in spring 2021. Students voluntarily complete the anonymous survey, answering questions about respiratory symptoms, smoking habits (both traditional and electronic cigarettes), and demographic information. The analysis focused on four subgroups, namely non-tobacco users, traditional cigarette smokers, e-cigarette users, and dual users, totaling 10,388 pupils aged 12–18 years, predominantly attending secondary technical and comprehensive schools. A total of 10,388 pupils participated in the study, 55.6% (5778) of whom were girls and 44.4% (4610) boys. Adolescents who admitted using both e-cigarettes and traditional cigarettes experienced more frequent episodes of cough during the day (39.70%) and at night (18.40%) compared to their peers in other groups. Chest discomfort, including pain and pressure, was also reported more often by adolescents who used e-cigarettes and traditional cigarettes concurrently (27.60%) compared to their peers in other groups. Chest pressure was experienced less commonly by non-smoking adolescents (14.40%) than by smokers (18.90%). Higher severity of cough during the day and at night was observed in the group of adolescents using traditional cigarettes and e-cigarettes concurrently compared to the other groups. The adolescents in the dual-user group experienced more severe dyspnea and wheezing compared to the other groups included in the comparison. The results of this study confirm the correlation between the occurrence of respiratory symptoms in adolescents who smoke cigarettes, adolescents who use e-cigarettes, and adolescents who are dual users. The respiratory symptoms occur most frequently and are the most severe in the group of adolescents who use e-cigarettes and traditional cigarettes

## 1. Introduction

In the recent years, we have observed a dynamic increase in the popularity of e-cigarettes (electronic cigarettes) among adolescents. In the studies conducted in Poland in 2016, it was noted that 28% of boys and 18.6% of girls aged 13–15 years declared using e-cigarettes [1]. What is more, 24% of adolescents declared using both traditional cigarettes and e-cigarettes concurrently [2]. E-cigarettes are the most commonly used tobacco product among adolescents in the US, and the observed increasing percentage of smokers is attributed to e-cigarettes [3]. The results from the 2019 and 2020 National Youth Tobacco Survey (NYTS), conducted by the Centers for Disease Control and Prevention (CDC) and the Food and Drug Administration (FDA), demonstrated that electronic cigarettes were the most frequently used tobacco product among high-school students (19.6%; 3.02 million) and junior high-school students (4.7%; 550 thousand) [4].

Thus, they have become a subject of intense scientific, medical, and social debate. The use of e-cigarettes, especially by adolescents, is still controversial in the context of public health studies [5,6]. Scientists frequently indicate adverse effects of e-cigarette use on the respiratory system in smokers, including adolescents and young adults. The studies involving adolescents demonstrated a correlation between e-cigarette use and increased incidence of bronchitis. Moreover, it was confirmed that both current and past use of e-cigarettes increases the probability of occurrence of the symptoms of bronchitis: cough and wheezing [7,8]. Similarly, the Yale Adolescent Survey Study demonstrated that e-cigarette use for six or more days within the previous 30 days increased the risk of occurrence of bronchitis [9]. In turn, researchers from California demonstrated that the risk of occurrence of bronchitis increases with higher frequency of e-cigarette use within the previous 30 days [10]. In a study performed in the years 2012–2013 in Hong Kong, it was found that electronic-cigarette use increases the risk of occurrence of chronic cough and bronchial secretions requiring evacuation [11]. There are also reports confirming a correlation between the use of e-cigarettes and the occurrence of asthma and its exacerbations. The results of studies performed in Florida [12] and Hawaii [13] confirmed the correlation between e-cigarette use and asthma in a group of young people. A study conducted among school students in South Korea evaluated the correlation between the use of e-cigarettes and the diagnosis of asthma within the previous 12 months. A comparison between current e-cigarette users and people who have never used electronic cigarettes demonstrated that the unadjusted odds ratio (OR) for asthma in the smokers was 2.36 (95% CI: 1.89–2.94). What is more, it was demonstrated that adolescents currently using e-cigarettes were at a higher risk of asthma exacerbation, represented by the number of days they were absent at school due to the symptoms of asthma compared to adolescents not using e-cigarettes [14].

The aim of this work was to evaluate the occurrence of respiratory symptoms and their severity in a group of adolescents using e-cigarettes and ones using both e-cigarettes and traditional cigarettes, so-called dual users.

## 2. Materials and Methods

In this study, we used a proprietary online survey to evaluate the occurrence of respiratory symptoms in Polish school students. The survey was planned for the 1st half of 2021, when classes in schools across Poland were held exclusively remotely. The survey questionnaire was shared through an online platform with schools’ Head Teachers to acquaint educators and teachers with its contents. Subsequently, Head Teachers throughout Poland were requested to facilitate the completion of the questionnaires by making them accessible to adolescents during school hours. The survey was administered during online classes, with the children participating from their homes. Children were not supervised while completing the survey to ensure complete anonymity of their responses.

The participants provided their voluntary consent to participate in the study by clicking the external link, which directed them to the online survey on the SurveyMonkey platform. The survey was completely anonymous. The following inclusion criteria were adopted: school students aged 12–18 years, in 6th–8th grade of the primary school, and 1st–4th grade of the secondary school who were willing to participate in the study. Exclusion criteria included incomplete or incorrectly filled questionnaires, participants younger than 12 or older than 18 years old, and children attending classes lower than the 6th grade of primary school. Furthermore, schools for children with disabilities were excluded from the study to prevent scenarios where children might need adult assistance to complete the survey. The survey was approved by the Bioethics Committee of the Medical University of Silesia in Katowice PCN/0022/KB1/63/21.

The survey included questions concerning the occurrence of respiratory symptoms, as well as traditional and electronic cigarette-smoking habits in adolescents. The survey also let us collect demographic data, such as gender, age, school type, population of their locality, level of their parents’ education, and tobacco-smoking status. The students were asked to choose and mark their answer regarding the incidence and severity of particular symptoms. The symptom severity was presented in the survey on a scale from 0 to 10, where 0 meant no symptoms and 10 meant symptoms interfering with their daily activities. Only current smokers were included in the analysis of the symptom occurrence and severity. This group consisted of adolescents who answered “yes” to the following questions: “Have you smoked traditional cigarettes within the last 30 days?” and/or “Have you used electronic cigarettes within the last 30 days?” Adolescents who gave a positive answer to both of these questions were classified as “dual users”. The statistical analysis was performed among four subgroups of school students: not using tobacco products (subgroup 1) (*n* = 8234), smoking only traditional cigarettes (subgroup 2) (*n* = 608), using only e-cigarettes (subgroup 3) (*n* = 879), and using traditional cigarettes and e-cigarettes concurrently (subgroup 4) (*n* = 667).

A total of 10,388 pupils participated in the study, 55.6% (5778) of whom were girls and 44.4% (4610) boys. The median age was 16.16 years (SD = 1.70). Considering the respondents’ schools, most of them attended secondary technical schools (4233 respondents, 40.7%). A smaller group attended secondary comprehensive schools (3376 respondents, 32.5%). Accordingly, 1988 respondents attended primary schools (19.1%), while only 791 students attended vocational schools (7.6%).

### Statistical Analysis

Statistical analyses were conducted utilizing the IBM SPSS Statistics 25 software package. Basic descriptive statistics were generated to summarize the central tendencies, dispersion, and distribution patterns of the data. To assess the normality of the data distributions, Kolmogorov–Smirnov tests were implemented. For comparing differences between two independent groups, the Mann–Whitney U tests were used, while the Kruskal–Wallis tests were applied for comparing differences among more than two groups. The Spearman’s ρ rank correlation analyses were performed to evaluate the strength and direction of associations between ordinal or non-normally distributed continuous variables. Additionally, χ^2^ tests with Bonferroni correction for multiple comparisons were utilized to examine the relationships between categorical variables. The conventional significance threshold of α = 0.05 was adopted to determine the statistical significance of the findings.

## 3. Results

The percentage of occurrence of the respective symptoms reported by the surveyed adolescents is presented in Table 1. The results for all symptoms were statistically significant (*p* < 0.001), but the effect size was low. In the latter stage of the study, a post hoc analysis of the results was performed using the χ^2^ test with Bonferroni correction for multiple comparisons.

Adolescents who admitted to using both e-cigarettes and traditional cigarettes experienced more frequent episodes of cough during the day (39.70%) and at night (18.40%) compared to their peers in other groups. Additionally, these symptoms were reported by more adolescents smoking traditional cigarettes than non-smokers and e-cigarette users; however, they occurred equally frequently in non-smokers and e-cigarette users.

In the dual-user group, dyspnea (28.80%), wheezing (24.60%), and sputum (40.50%) occurred more commonly than in other groups. Moreover, these symptoms occurred significantly less frequently in adolescents who did not use e-cigarettes and traditional cigarettes than in the groups using traditional cigarettes or e-cigarettes only. Dyspnea and wheezing occurred equally frequently in traditional cigarette smokers and e-cigarette users, whereas sputum was reported more frequently by traditional cigarette smokers (33.70%) and e-cigarette users (26.60%).

The adolescents in the dual-user group indicated trouble catching breath during the day and difficulty breathing at night more commonly than adolescents in other groups. Non-smoking school students reported trouble catching breath during the day significantly less frequently than traditional cigarette smokers (*p* < 0.001). Chest discomfort, including pain and pressure, was also reported more often by adolescents, who used e-cigarettes and traditional cigarettes concurrently (27.60%) compared to their peers in other groups. Chest pressure was experienced less commonly by non-smoking adolescents (14.40%) than by smokers (18.90%).

The next analysis concerned the incidence of symptoms in adolescents smoking traditional cigarettes and the ones who did not smoke (regardless of whether they used e-cigarettes or not). The results of this analysis are presented in Table 2. Cough during the day, cough at night, dyspnea, difficulty breathing during the day, difficulty breathing during sleep, wheezing, chest pain, chest pressure, and sputum were more frequently reported in the group of children smoking traditional cigarettes and turned out to be statistically significantly more common in children smoking traditional cigarettes (*p* < 0.001), but the effect size was low.

The following comparative analysis concerned the incidence of symptoms in adolescents using e-cigarettes and the ones who did not use this product (regardless of whether they smoked traditional cigarettes or not). The results, of which nine were statistically significant (*p* < 0.001), are presented in Table 3. Cough during the day, cough at night, dyspnea/difficulty breathing, trouble catching breath during the day, difficulty breathing during sleep, wheezing, chest pain, chest pressure, and sputum were more frequently reported in the group of adolescents using e-cigarettes. However, the effect size was low.

In the end, we checked whether the increased frequency of occurrence of certain respiratory symptoms is significantly correlated with e-cigarette and traditional cigarette use. Thus, we performed a series of Kruskal–Wallis tests in the four groups of adolescents. The obtained results are presented in Table 4, and all turned out to be statistically significant. Due to this fact, we also conducted a series of post hoc analyses, along with the application of the Dunn–Šidák correction.

A higher severity of cough during the day and at night was observed in the group of adolescents using traditional cigarettes and e-cigarettes concurrently compared to the other groups. The smoking adolescents reported a more severe cough during the day and at night compared to both non-smokers and adolescents using e-cigarettes only. The adolescents in the dual-user group experienced more severe dyspnea and wheezing compared to the other groups included in the comparison. The symptoms were less severe in non-smokers than in adolescents smoking traditional cigarettes or using e-cigarettes only. Analyzing the occurrence of symptoms related to trouble catching breath during the day and at night, it was found that, in adolescents using e-cigarettes and smoking traditional cigarettes concurrently, these symptoms were more severe compared to the other groups. Non-smoking adolescents experienced breathing difficulties less than smokers or e-cigarette users. Chest pain and pressure was found to be more severe in the group of adolescents classified as dual users.

Also, in the case of sputum, the adolescents using e-cigarettes and smoking traditional cigarettes reported the most severe symptoms. In addition, the more severe discomfort associated with sputum was reported by traditional cigarette smokers than e-cigarette users.

## 4. Discussion

In the recent years, we have observed an increase in the popularity of e-cigarettes as an alternative to traditional tobacco products. The effect of this trend on public health, especially in the context of the health of children and adolescents, has become a subject of an increased interest among scientists and the public. Adolescents constitute a group of special concern when it comes to the use of tobacco products and e-cigarettes because they tend to experiment with various forms of stimulants, including e-cigarettes.

There are studies regarding the effect of smoking traditional cigarettes on respiratory health. Thus, considerable attention is also paid to potential health hazards associated with the use of e-cigarettes by adolescents. There is an increasing number of studies analyzing the correlation between e-cigarette use and various respiratory symptoms in children and adolescents.

In various studies, the authors use different variables to compare the group of e-cigarette users with users of other nicotine-delivery products, and they also use various classifications and comparisons in terms of frequency or history of use of various tobacco products. This is a common topic in the scientific literature because there is no consensus on how to measure the effects of the use of e-cigarettes and tobacco products.

In this work, we adopted a simple and practical division of adolescents into four study groups, according to the use of tobacco products: non-smokers, traditional cigarette smokers, e-cigarette users, and dual users. The non-smoker group included adolescents who had never used any tobacco products. The traditional cigarette-smoker group included adolescents who regularly smoked conventional cigarettes, while the group of e-cigarette users consisted of adolescents who used e-cigarettes without using traditional cigarettes. The dual-user group included adolescents who used both e-cigarettes and traditional cigarettes. By making this division, we could study the differences between these groups regarding the respiratory symptoms more accurately and evaluate the potential health hazard associated with various forms of tobacco-product use by children and adolescents.

Our study stands out from other scientific works due to the special focus on the issue of the concurrent use of both traditional cigarettes and e-cigarettes by children and adolescents. Although there is an increasing number of studies concerning the effect of every form of smoking on the respiratory system, not much attention is devoted to the concurrent use of both of these products in the same age group. We conducted a detailed analysis of the incidence of respiratory symptoms in the study group, highlighting the need to understand and evaluate the effect of dual use of traditional cigarettes and e-cigarettes on the health of children and adolescents.

In turn, the authors of the PATH study performed an analysis of the effect of e-cigarette use on the respiratory system in relation to the duration of smoking. In stages 3 and 4 of the PATH study, which took place between October 2015 and January 2018, the occurrence of wheezing in the previous year was assessed in adolescents aged 12–17 years who declared smoking e-cigarettes in the previous 7 days, 30 days, and one year, and a group of adolescents who claimed that they had never used e-cigarettes. In the study group, 8.1% surveyed adolescents confirmed experiencing wheezing within the previous 12 months. Electronic-cigarette use was associated with a relatively high odds of wheezing in the last 12 months in the unadjusted model (OR, 1.74; 95% CI, 1.22–2.48); it was slightly lower in those who had smoked in the previous 30 days (OR, 1.66; 95% CI, 0.86–3.21) and in the previous 7 days (OR, 1.31; 95% CI, 0.63–2.69). In turn, in the adjusted model, the correlation between the use of e-cigarettes and wheezing was significantly lower in adolescents who used ENDC in the previous year (aOR, 1.37; 95% CI, 0.91–2.05), in the previous 30 days (aOR, 1.35; 95% CI, 0.63–2.88), and in the previous 7 days (aOR, 0.74; 95% CI, 0.28–1.97) [15].

In the subsequent study, the researchers divided adolescents into similar groups, as we did in our work. The data come from the 4th and 5th stages of the PATH study [16]. The results were analyzed to examine the correlation between tobacco smoking and the use of electronic nicotine-delivery systems, including dual users, and the incidence of respiratory diseases. The study demonstrated that concurrent use of traditional and electronic nicotine products was associated with the risk of respiratory diseases in adolescents. Exclusive use of traditional cigarettes (aOR = 2.10, 95% CI = 1.17–3.77) or e-cigarettes (aOR = 1.03, 95% CI = 0.49–2.16), and concurrent use of both products (aOR = 2.02, 95% CI = 1.20–3.40) were associated with a greater likelihood of an increased risk of respiratory diseases compared to not using any nicotine-delivery products [16].

Our results are in accordance with the results obtained in the 4th and 5th stage of the PATH study; all the analyzed respiratory symptoms were most prevalent in the group of adolescents using traditional cigarettes and e-cigarettes concurrently.

In order to evaluate the effect of e-cigarette use on respiratory health using the 2012–2013 Global Tobacco Survey, a cross-sectional study was performed in Hong Kong among 45,128 adolescents aged 12–17 years [11]. The use of e-cigarettes was determined based on a survey conducted among school students, who declared themselves non-smokers or current, experimental, or past e-cigarette users. That study demonstrated a significant correlation between the use of e-cigarettes and increased risk of chronic cough and sputum. Respiratory symptoms were defined as cough or sputum persisting for 3 consecutive months within the previous 12 months. A detailed data analysis demonstrated that 33.9% of e-cigarette users reported these symptoms compared to 19.4% of non-users. The probability (OR) of the occurrence of these symptoms in e-cigarette users was 2.13 (95% CI: 1.82–2.48, *p* < 0.01), and the adjusted OR was 1.28 (95% CI: 1.06–1.56, *p* < 0.05). The study demonstrated a significant correlation between respiratory symptoms and the use of e-cigarettes compared to people who never used e-cigarettes (OR: 2.09, 95% CI: 1.27–3.44, *p* < 0.01; adjusted OR: 2.06, 95% CI: 1.24–3.42, *p* < 0.01), incidental smokers (OR: 1.45, 95% CI: 1.19–1.78; adjusted OR: 1.39, 95% CI: 1.14–1.7, *p* < 0.01), and former smokers (OR: 1.46, 95% CI: 1.07–2.00 adjusted OR: 1.4; 95% CI: 1.02–1.91, *p* < 0.05) [11].

In 2014, the Southern California Children’s Health Study performed among 11th and 12th grade students demonstrated that the risk of occurrence of chronic cough, sputum, or bronchitis increased almost twofold among both former and current e-cigarette users. However, no statistically significant correlation was found between the use of e-cigarettes and the occurrence of wheezing. The study was conducted in schools among 502 school students aged 16–18 years, using a questionnaire. The use of e-cigarettes was defined in a similar way as past or current e-cigarette use. Additionally, there was also a control group consisting of students who had never used e-cigarettes. The analyzed respiratory symptoms included symptoms associated with chronic bronchitis; the study participants were asked whether they had experienced daily cough, rhinitis, or sputum for three consecutive months. The symptoms of bronchitis were reported by 17.3% of subjects who had never used e-cigarettes, 24.2% former users, and 35.5% current users. A correlation was demonstrated between the symptoms of bronchitis and the use of e-cigarettes in the past (odds ratio (OR): 1.85; 95% confidence interval (CI): 1.37–2.49) and current users (OR: 2.02; 95% CI: 1.42 –2.88). The risk of occurrence of bronchitis symptoms increased with a higher frequency of e-cigarette use (OR: 1.66; 95% CI: 1.02–2.68 for 1–2 days) (OR: 2.52; 95% CI: 1.56–4.08 for 3 days or longer) compared to study participants who had never used e-cigarettes [17].

In contrast to other authors, we distinguished two different types of coughs in our survey: daytime cough and nighttime cough. In terms of the occurrence of cough and sputum, the results of our study correspond with those of the study performed among adolescents in Hong Kong and California. In our study, 24.8% adolescents using e-cigarettes and 39.7% dual users reported cough during the day, while 7.8% and 18.4%, respectively, reported cough at night. Analyzing the accumulation of sputum, we demonstrated that the symptom occurs in 26.6% of adolescents using e-cigarettes and 40.5% of dual users. Adolescents in the dual-user group experienced a more frequent and more severe cough during the day and sputum accumulation than those in the other groups.

In 2015–2016, Cherian et al. conducted a study to assess the correlation between vaping and the occurrence of respiratory symptoms among adolescents following a significant increase in national rates of ENDS use among youth in the US. Using the data from the PATH study, constituting a representative dataset for the US, they observed that adolescents who reported using ENDS in the past 12 months, compared with their peers who did not use ENDS in the past 12 months, had a greater overall risk of wheezing. They also found that the risk of dry cough at night was higher in school students using e-cigarettes within the previous 12 months than in non-smokers. The results partly confirm the hypothesis that the use of ENDS by adolescents is associated with respiratory symptoms, including cough and wheezing [7].

Our results are in line with those obtained by Cheriant et al. regarding the occurrence of wheezing. Adolescents who do not use any tobacco products reported wheezing less frequently than the ones smoking only traditional cigarettes or only e-cigarettes. This symptom was reported more frequently and was more severe in adolescents in the dual-user group than in the other groups. In contrast to Cherian et al., the difference we found between the occurrence of cough at night in adolescents using e-cigarettes and the ones not using any tobacco products was not statistically significant.

The authors of the LUIS study, conducted in 2013–2016 in the Swiss canton of Zurich, analyzed the occurrence of respiratory symptoms in children and adolescents aged 6–17 years who used various tobacco products, including traditional cigarettes, e-cigarettes, and shisha. The participants self-reported the occurrence of such symptoms as a cough not associated with the common cold, more frequent than in their peers, rhinitis, dry mouth after waking up, dyspnea, spontaneous wheezing, and wheezing after physical effort. The results of the study confirmed that the use of any tobacco products by adolescents was associated with a higher incidence of respiratory symptoms. Children and adolescents who smoked cigarettes or shisha or used e-cigarettes often or only occasionally reported a higher incidence of respiratory symptoms within the previous 12 months than the ones who did not use these products. The smokers also experienced other respiratory symptoms more frequently, including rhinitis not associated with the common cold and dry mouth after waking up. Additionally, they reported dyspnea, unprovoked wheezing, and wheezing associated with physical effort. Wheezing and wheezing associated with physical effort occurred more frequently in adolescents who smoked often or occasionally compared to the ones who did not smoke. The results demonstrated that the use of tobacco products may constitute a risk factor for respiratory health in adolescents and highlighted the need to implement preventive measures among the youth [18].

The results of our study turned out to be consistent with the LUIS study, with such symptoms as cough, dyspnea, wheezing, and sputum accumulation occurring more frequently in adolescents either smoking traditional cigarettes or using e-cigarettes, as well as dual users, than in their non-smoking peers. Dyspnea and trouble catching breath were reported more frequently in the dual-user group (28.8%), traditional cigarette smokers (22.9%), and e-cigarette users (18.7%). These symptoms were reported by 14.3% of non-smoking adolescents.

In the study by King et al., the authors compared the symptoms reported by adolescents using e-cigarettes, traditional cigarettes, and other tobacco products (water pipe, small cigars or cigarillos, and other types of tobacco) in the previous 30 days compared to adolescents who did not declare the use of tobacco products in the same 30-day period. The study identified the probability of an increased incidence of symptoms in the case of using more than one tobacco product (polytobacco use) compared to sticking to only one product or not using any form of tobacco at all. The evaluated symptoms included cough, dizziness or lightheadedness, headache or migraine, dry mouth or oral/pharyngeal irritation, dyspnea, and disturbance or loss of taste, and more than a half (63.3%) of young people who were using or had ever used e-cigarettes reported the occurrence of one or more symptoms associated with e-cigarette use (in the range from 0 to 5 symptoms). On average, the adolescents reported 1.4 (SE = 0.2) symptoms, including cough (42.3%), dizziness or lightheadedness (31.5%), headache or migraine (25.4%), dry mouth or oral/pharyngeal irritation (14.9%), and dyspnea (13.7%; see Table 2). Few adolescents reported disturbance or loss of taste (3.5%) or other symptoms (5.7%), including nausea, dry eyes, earache, and chest pressure. The study participants who declared e-cigarette use within the previous 30 days reported headache or migraine more frequently than the ones who did not use e-cigarettes in the same 30-day period (43.8% vs. 14.6%, *p* = 0.002). Similarly, the participants who declared using e-cigarettes within the previous 30 days more commonly attributed their symptoms to the use of e-cigarettes (86.0% vs. 58.6%, *p* = 0.001) and dyspnea (33.8% vs. 9.5%, *p* = 0.03). Moreover, the participants who declared the use of another tobacco product within the previous 30 days reported a higher incidence of dyspnea caused by e-cigarette use (31.6% vs. 8.2%, *p* = 0.01). Finally, the participants who declared using more than one product within the previous 30 days reported a higher incidence of headache or migraine than the ones who had not used any tobacco products in the same 30-day period (39.1% vs. 11.7%).

In the case of every group of tobacco product (e-cigarettes, traditional cigarettes, and other products), users who had used any product within the previous 30 days reported the occurrence of at least one symptom more frequently than non-smokers, including headache or migraine and dyspnea. What is more, the participants who had used e-cigarettes within the previous 30 days reported symptoms associated with their use more frequently than those who had not. It is possible that the adolescents using other tobacco products experience more symptoms when using e-cigarettes. It is also possible that the adolescents using other tobacco products may attribute the symptoms associated with the use of other products to e-cigarettes [19].

In our study population of adolescents, the symptom most frequently reported by e-cigarette users was sputum accumulation—26.6%. The second most prevalent symptom in this group was cough during the day—24.8%. Our results obtained in the group of dual users, who may be considered polytobacco users, are in line with the results obtained by King et al.

Despite the extensive scientific literature available on e-cigarette use, a majority of these studies predominantly examine adult populations. This article is noteworthy due to its focus on the pediatric population, specifically targeting children aged 12–18 years. The investigation addresses a significant gap in the literature pertaining to Poland and Eastern Europe, where there is a shortage of research on respiratory symptoms in adolescents using e-cigarettes. Moreover, our study focuses on a specific subset of dual users—children who use both e-cigarettes and traditional cigarettes, what is rarely seen in Eastern European publications. The data collected include a large cohort of children across Poland, providing a comprehensive analysis.

## 5. Limitations

Our project is strengthened by a robust design featuring a large sample of school-aged adolescents in Poland. It significantly contributes to the literature on youth tobacco use by thoroughly examining patterns of combustive cigarette use, e-cigarette use, and dual product use among adolescents.

Online surveys are often limited by the lack of internet access or by the selective interests and browsing habits of the respondents. However, in this case, we managed to mitigate this issue since, during the survey period, all the students from the participating schools had access to internet-enabled devices due to the remote learning. Additionally, our survey was sent directly to each student through the schools that expressed a willingness to participate in the project.

This study has several limitations. First, our study targeted school students. This is a selected group of individuals, and the results of our study cannot be generalized to the whole population. Secondly, our study relied on adolescent self-report, which is limited by their willingness and ability to report honestly. We did not provide a correlation analysis regarding age, gender, and Body Mass Index (BMI), which could provide a more nuanced understanding of the results. In addition, the study did not collect detailed data on the number of cigarettes smoked or the number of puffs taken per day, which may affect the severity of respiratory symptoms.

## 6. Conclusions

The results of this study confirm the correlation between the occurrence of respiratory symptoms in adolescents who smoke cigarettes, adolescents who use e-cigarettes, and adolescents who are dual users. The respiratory symptoms occur most frequently and are the most severe in the group of adolescents who use e-cigarettes and traditional cigarettes. By highlighting the aspect of dual use, we have attempted to make an important contribution to the development of knowledge on the effect of smoking on the health status of the young generation and underline the need for intervention aimed at reducing this phenomenon and protecting public health.

Due to the dynamics of the tobacco product market and the evolving forms of tobacco use, it is necessary to continuously monitor and evaluate the effect of e-cigarette use on the health of young people.

## Figures and Tables

**Table 1:** The occurrence of specific symptoms depending on the use of e-cigarettes and traditional cigarettes.

	Do Not Use Tobacco Products (*n* = 8234)	Smoke Traditional Cigarettes (*n* = 608)	Use E-Cigarettes (*n* = 879)	Use Traditional Cigarettes and E-Cigarettes (*n* = 667)	
Cough during the day	1835 (F = 1085; M = 750)	200 (F = 108; M = 92)	218 (F = 107; M = 111)	265 (F = 141; M = 124)	χ^2^(3) = 129.23 *p* < 0.001 *V* = 0.11
	22.30%	32.90%	24.80%	39.70%	
Cough at night	638 (F = 387; M = 251)	83 (F = 45; M = 38)	69 (F = 33; M = 36)	123 (F = 63; M = 60)	χ^2^(3) = 107.53 *p* < 0.001 *V* = 0.10
	7.70%	13.70%	7.80%	18.40%	
Shortness of breath or breathing difficulties	1177 (F = 843; M = 334)	139 (F = 87; M = 52)	164 (F = 87; M = 77)	192 (F = 116; M = 76)	χ^2^(3) = 124.20 *p* < 0.001 *V* = 0.11
	14.30%	22.90%	18.70%	28.80%	
Trouble catching breath during the day	982 (F = 709; M = 273)	101 (F = 69; M = 32)	120 (F = 73; M = 47)	172 (F = 106; M = 66)	χ^2^(3) = 109.96 *p* < 0.001 *V* = 0.10
	11.90%	16.60%	13.70%	25.80%	
Breathing difficulties during sleep	496 (F = 329; M = 367)	37 (F = 22; M = 15)	57 (F = 26; M = 31)	97 (F = 52; M = 45)	χ^2^(3) = 72.84 *p* < 0.001 *V* = 0.08
	6.00%	6.10%	6.50%	14.50%	
Wheezing sounds in the chest	428 (F = 279; M = 149)	89 (F = 51; M = 38)	102 (F = 44; M = 58)	164 (F = 91; M = 73)	χ^2^(3) = 407,73 *p* < 0.001 *V* = 0.20
	5.20%	14.60%	11.60%	24.60%	
Chest pain	1273 (F = 899; M = 374)	119 (F = 72; M = 47)	152 (F = 84; M = 68)	192 (F = 108; M = 84)	χ^2^(3) = 82.91 *p* < 0.001 *V* = 0.09
	15.50%	19.60%	17.30%	28.80%	
Tightness in the chest	1182 (F = 849; M = 333)	115 (F = 72; M = 43)	154 (F = 86; M = 68)	184 (F = 104; M = 80)	χ^2^(3) = 89.21 *p* < 0.001 *V* = 0.09
	14.40%	18.90%	17.50%	27.60%	
Bronchial secretions requiring coughing out	1534 (F = 852; M = 682)	205 (F = 115; M = 90)	234 (F = 107; M = 127)	270 (F = 135; M = 135)	χ^2^(3) = 249.15 *p* < 0.001 *V* = 0.16
	18.60%	33.70%	26.60%	40.50%	

**Table 2:** The occurrence of specific symptoms depending on the use of traditional cigarettes.

	Do Not Smoke Traditional Cigarettes (*n* = 9113)	Smoke Traditional Cigarettes (*n* = 1275)	χ^2^	Odds Ratio (95% *CI*)
Cough during the day	2053	465	χ^2^(1) = 118.40 *p* < 0.001 *V* = 0.11	1.97%
	22.50%	36.50%		(1.74–2.24)
Cough at night	707	206	χ^2^(1) = 98.42 *p* < 0.001 *V* = 0.10	2.29%
	7.80%	16.20%		(1.94–2.71)
Shortness of breath or breathing difficulties	1341	331	χ^2^(1) = 104.74 *p* < 0.001 *V* = 0.10	2.03%
	14.70%	26.00%		(1.77–2.33)
Trouble catching breath during the day	1102	273	χ^2^(1) = 84.58 *p* < 0.001 *V* = 0.09	1.98%
	12.10%	21.40%		(1.71–2.30)
Breathing difficulties during sleep	553	134	χ^2^(1) = 35.73 *p* < 0.001 *V* = 0.06	1.82%
	6.10%	10.50%		(1.49–2.22)
Wheezing sounds in the chest	530	253	χ^2^(1) = 315.78 *p* < 0.001 *V* = 0.17	4.01%
	5.80%	19.80%		(3.41–4.72)
Chest pain	1425	311	χ^2^(1) = 61.60 *p* < 0.001 *V* = 0.08	1.74%
	15.60%	24.40%		(1.51–2.00)
Tightness in the chest	1336	299	χ^2^(1) = 65.17 *p* < 0.001 *V* = 0.08	1.78%
	14.70%	23.50%		(1.55–2.06)
Bronchial secretions requiring coughing out	1768	475	χ^2^(1) = 210.60 *p* < 0.001 *V* = 0.14	2.47%
	19.40%	37.30%		(2.18–2.80)

**Table 3:** The occurrence of specific symptoms depending on the use of e-cigarettes.

	Do Not Use E-Cigarettes (*n* = 8842)	Use E-Cigarettes (*n* = 1546)		Odds Ratio (95% *CI*)
Cough during the day	2035	483	χ^2^(1) = 48.50 *p* < 0.001 *V* = 0.07	1.52%
	23.00%	31.20%		(1.35–1.71)
Cough at night	721	192	χ^2^(1) = 29.86 *p* < 0.001 *V* = 0.05	1.60%
	8.20%	12.40%		(1.35–1.89)
Shortness of breath or breathing difficulties	1316	356	χ^2^(1) = 64.62 *p* < 0.001 *V* = 0.08	1.71%
	14.90%	23.00%		(1.50–1.95)
Trouble catching breath during the day	1083	292	χ^2^(1) = 50.51 *p* < 0.001 *V* = 0.07	1.67%
	12.20%	18.90%		(1.45–1.92)
Breathing difficulties during sleep	533	154	χ^2^(1) = 32.96 *p* < 0.001 *V* = 0.06	1.73%
	6.00%	10.00%		(1.43–2.08)
Wheezing sounds in the chest	517	266	χ^2^(1) = 243.60 *p* < 0.001 *V* = 0.15	3.35%
	5.80%	17.20%		(2.85–3.92)
Chest pain	1392	344	χ^2^(1) = 40.04 *p* < 0.001 *V* = 0.06	1.53%
	15.70%	22.30%		(1.34–1.75)
Tightness in the chest	1297	338	χ^2^(1) = 51.36 *p* < 0.001 *V* = 0.07	1.63%
	14.70%	21.90%		(1.42–1.86)
Bronchial secretions requiring coughing out	1739	504	χ^2^(1) = 130.00 *p* < 0.001 *V* = 0.11	1.98%
	19.70%	32.60%		(1.76–2.22)

**Table 4:** The severity of specific symptoms depending on the use of e-cigarettes and traditional cigarettes.

	Do Not Use Tobacco Products (*n* = 8234)		Smoke Traditional Cigarettes (*n* = 608)		Use E-Cigarettes (*n* = 879)		Use Traditional Cigarettes and E-Cigarettes (*n* = 667)			
	*M*	*SD*	*M*	*SD*	*M*	*SD*	*M*	*SD*	*F*	*p*
Cough during the day	0.45%	1.12%	0.75%	1.42%	0.60%	1.45%	1.23%	2.24%	160.14%	0.001%
Cough at night	0.16%	0.77%	0.27%	0.90%	0.22%	1.01%	0.69%	1.97%	115.44%	0.001%
Shortness of breath or breathing difficulties	0.36%	1.19%	0.52%	1.28%	0.50%	1.41%	1.07%	2.33%	134.94%	0.001%
Trouble catching breath during the day	0.28%	1.03%	0.43%	1.24%	0.38%	1.27%	0.96%	2.27%	121.35%	0.001%
Breathing difficulties during sleep	0.14%	0.79%	0.13%	0.68%	0.21%	1.05%	0.59%	1.91%	77.29%	0.001%
Wheezing sounds in the chest	0.13%	0.74%	0.40%	1.22%	0.30%	1.10%	0.83%	2.09%	416.81	0.001%
Chest pain	0.38%	1.21%	0.57%	1.52%	0.43%	1.35%	1.02%	2.27%	93.87%	0.001%
Tightness in the chest	0.35%	1.16%	0.53%	1.50%	0.45%	1.38%	1.00%	2.32%	99.03%	0.001%
Bronchial secretions requiring coughing out	0.46%	1.36%	1.10%	2.13%	0.76%	1.74%	1.46%	2.56%	287.12%	0.001%

## Data Availability

The original contributions presented in the study are included in the article; further inquiries can be directed to the corresponding author.
